# Distinct Roles of Medial Prefrontal Cortex Subregions in the Consolidation and Recall of Remote Spatial Memories

**DOI:** 10.1523/ENEURO.0192-24.2024

**Published:** 2024-10-16

**Authors:** Eleonora Centofante, Mattia Santoboni, Elena L. J. Mombelli, Arianna Rinaldi, Andrea Mele

**Affiliations:** Department of Biology and Biotechnology ‘C. Darwin’, Centre for Research in Neurobiology ‘D.Bovet’, Sapienza University of Rome, Rome I-00185, Italy

**Keywords:** anterior cingulate cortex, consolidation, infralimbic cortex, Morris water maze, prelimbic cortex

## Abstract

It is a common belief that memories, over time, become progressively independent of the hippocampus and are gradually stored in cortical areas. This view is mainly based on evidence showing that prefrontal cortex (PFC) manipulations impair the retrieval of remote memories, while hippocampal inhibition does not. More controversial is whether activity in the medial PFC is required immediately after learning to initiate consolidation. Another question concerns functional differences among PFC subregions in forming and storing remote memories. To address these issues, we directly contrasted the effects of loss-of-function manipulations of the anterior cingulate cortex (aCC) and the ventromedial PFC, which includes the infralimbic (IL) and prelimbic (PL) cortices, before testing and immediately after training on the ability of CD1 mice to recall the hidden platform location in the Morris water maze. We injected an AAV carrying the hM4Di receptor into the PL–IL or aCC. Interestingly, pretest administrations of clozapine-N-oxide (CNO; 3 mg/kg) revealed that the aCC, but not the PL–IL, was necessary to recall remote spatial information. Furthermore, systemic post-training administration of CNO impaired memory recall at remote, but not recent, time points in both groups. These findings revealed a functional dissociation between the two prefrontal areas, demonstrating that both the PL–IL and the aCC are involved in early consolidation of remote spatial memories, but only the aCC is engaged in their recall.

## Significance Statement

Contrasting the two main components of the prefrontal cortex, the anterior cingulate (aCC) and the ventro medial component, which includes the prelimbic and the infralimbic areas (PL–IL), we investigated their respective contributions to the recall of remote spatial memory and the consolidation of both recent and remote memories. The results demonstrated an interesting dissociation between these regions. Loss-of-function manipulation of the aCC, but not the PL–IL, impaired the recall of remote spatial memory. However, both subdivisions contributed to the consolidation of remote spatial memory. These findings provide new insight into the circuit dynamic involved in the formation of stable remote memories, suggesting a regulatory role of the PL–IL in selecting relevant information from storage.

## Introduction

Memory traces that outlast the initial stages and represent events that occurred from days to years before are defined as remote memories (Sadeh et al., 2014). Theories relevant to the underpinning of remote memories agree on the central role of the hippocampus in the initial stages of memory formation (Scoville and Milner, 1957; Sadeh et al., 2014). Nevertheless, the common view is that over weeks, the role of the hippocampus progressively degrades, and cortical structures become increasingly important in the stabilization of memory (Frankland and Bontempi, 2005). Two lines of evidence support this hypothesis. Firstly, mapping neuronal activity at early and late stages after training demonstrates increased (^14^C)2-deoxyglucose uptake and immediate early gene (IEG) expression in cortical areas after remote, but not recent memory, retrieval; secondly, loss-of-function manipulations of the prefrontal cortex (PFC) impair expression of memory at remote, but not recent, time points (Bontempi et al., 1999; Maviel et al., 2004; Teixeira et al., 2006; Lesburguères et al., 2011; Lopez et al., 2012; Wartman et al., 2014). Collectively, these observations have given rise to the view that the role of the neocortex is limited to the late stages of memory processing to subserve remote memory storage and recall. However, although this hypothesis has been convincingly demonstrated for aversively motivated tasks, the extent to which it applies to spatial memories remains more controversial (Martin et al., 2005; Sutherland and Lehmann, 2011). Moreover, most studies investigating the engagement of the PFC in remote memory have primarily focused on recall, while much less is known about its potential involvement in the consolidation process, which could provide relevant insights into the temporal dynamics of its participation.

The relative contribution of the different components of the PFC in remote spatial memory is also a key issue. The PFC constitutes a heterogeneous brain region, and, based on recent connectivity studies, it has been suggested that at least two subdivisions of the PFC can be recognized, the dorsomedial PFC (anterior cingulate cortex, aCC) and the ventromedial PFC, which includes the prelimbic (PL) and infralimbic (IL) cortices (Heidbreder and Groenewegen, 2003; Uylings et al., 2003; Laubach et al., 2018; Le Merre et al., 2021). Although the connectivity of the two subdivisions is not completely differentiated, it has been suggested that the aCC and the PL–IL can be dichotomized based on their sensory motor and limbic hodological profiles (Heidbreder and Groenewegen, 2003; Uylings et al., 2003; Le Merre et al., 2021). In particular, the PL–IL subdivision is defined by its limbic connectivity, which encompasses the hippocampal formation, the amygdala, and the reciprocal connectivity with the ventral tegmental area (Parent et al., 2010; Kim et al., 2017). On the other hand, the aCC is distinguished by its reciprocal connections with sensory and motor cortical regions (Groenewegen et al., 1987; Le Merre et al., 2021), the striatum, and by its thalamic inputs (Vertes, 2002). This anatomical subdivision is accompanied by functional distinctions in the processing of spatial information (Sutherland et al., 1988; Kolb et al., 1994) as well as memory recall (Frankland et al., 2004; Maviel et al., 2004). Particularly relevant to our study is the finding that IEG expression demonstrates increased neural activity in both components of the PFC when recall occurs at remote time points, while causal studies reveal task-dependent effects on remote memory recall of the two PFC components (Frankland et al., 2004; Maviel et al., 2004; Lopez et al., 2012). Thus, it is difficult to draw clear conclusions on the specific role of the PL–IL and the aCC in the formation and storage of spatial memory traces.

In this study, we first asked whether the two subdivisions of the PFC could play differential roles in the recall of remote spatial memory by using chemogenetic tools, demonstrating a functional difference between the PL–IL and the aCC. In light of the difference outlined in the recall of remote spatial memory, we asked whether the same differences could also be found in the consolidation. Thus, next, we investigated the role of the two components of the PFC in the consolidation of recent and remote spatial memory. Interestingly, in this case, inhibition of both the PL–IL and the aCC had similar effects, impairing the ability to consolidate the platform location at remote time points.

## Materials and Methods

### Subjects

Experiments were conducted on CD1 male mice (*Mus musculus*; Charles River Laboratories), 7–9 weeks old at the time of surgery. Animals were housed in groups of four in standard cages (26.8 × 21.5 × 14.1 cm) with food and water *ad libitum* under a 12 h (7:00 A.M.–7:00 P.M.) light/dark cycle. The humidity and temperature of the room were constant (21 ± 1°C). Every experimental procedure was conducted during the light period (9:00 A.M.–5:00 P.M.). The maximum effort was made to minimize animal suffering. Procedures were conducted under Authorization Number 450-2018 from the Italian Ministry of Health, according to Italian (DL 116/92) and European laws on the use of animals in research and NIH guidelines on animal care.

### Stereotaxic surgery

Mice were deeply anesthetized with 3% isoflurane (Isovet; Piramal Healthcare) and secured on the stereotaxic apparatus (David Kopf Instruments) with a mouse adapter and lateral ear bars. Following craniotomies, glass micropipettes were lowered in the PL–IL or in the aCC, according to the mouse brain atlas (Franklin and Paxinos, 1997). The following coordinates were used: for the PL–IL, AP, +1.7 mm; ML, ±0.3 mm; and DV, −2.3 mm, and for the aCC, AP, +0.8 mm; ML, ±0.2 mm; and DV, −1.8 mm, relative to the bregma. Viral delivery was performed connecting the glass micropipette to a syringe. Once the micropipette reached the correct placement, it was left in place for 1 min before starting the injection. Adeno-associated viral vector (AAV2) was used to express the mCherry fluorescent protein and human influenza hemagglutinin-tagged hM4Di under the human synapsin1 promoter [pAAV-hSyn-hM4D(Gi)-mCherry; Addgene #50475-AAV2]. The volume injected was always 0.3 μl, for each hemisphere, over 2 min. After each injection, the pipette was left in place for 5 additional minutes to allow diffusion. After surgery, the wound was disinfected with Betadine, and the mice were placed in a recovery cage at a controlled temperature with the use of a thermal plate, before returning to the animal room for recovery.

### Behavioral apparatus and procedure

The apparatus used for the Morris water maze (MWM) consisted of a circular pool (110 cm diameter and 40 cm high), filled up to 5 cm from the edge with black-colored (Giotto) water (22 ± 1°C) as previously described (Ferretti et al., 2010; Centofante et al., 2023). Black curtains surrounded the pool, and several visual cues were attached on them at a distance of ∼50 cm from the pool. Four starting positions were located equidistantly around the edge of the pool wall, virtually dividing the swimming pool into four equal quadrants. During the training, a circular black goal platform (10 cm of diameter), covered with wire mesh to avoid slipping, was positioned 18 cm from the wall. The apparatus was illuminated by a white diffuse light.

The procedure consisted of three different phases: a familiarization phase (on Day 1), a training phase (on Day 2), and one or two probe tests, 30 d or 24 h and 30 d after the last training session, respectively, for the recall or the consolidation experiments. Before each phase, animals were placed in isolation cages, without food or water, for 30 min. Each session during familiarization and training consisted of three 60 s trials, while the probe test consisted of a single 60 s trial. During the familiarization phase, mice were submitted to three trials (intertrial interval, 20 s) during which the extra maze cues were removed and the platform was made visible (1 cm over the water). The training phase consisted of a single day massed procedure of six consecutive sessions (intersession interval, 10–15 min) of three trials (intertrial interval, 30 s). During the probe test, the platform was removed, and mice were allowed a 60 s search for the platform starting from the center of the pool. At the end of the behavioral procedures, mice were perfused to verify the viral expression. All trials were recorded by a camera located over the pool, and they were acquired and analyzed using an automated video tracking system (ANY-maze 5.0, Stoelting).

### Drug injection

Clozapine-N-oxide (CNO; HelloBio) 3 mg/kg was dissolved in saline 0.9% and made fresh every other week. For the experiment that intended to investigate the role of PL–IL or aCC in the consolidation of remote spatial memory, CNO was administered immediately after the last training session; for experiments that intended to investigate the role of PL–IL or aCC in the retrieval of remote spatial memory, CNO was administered 30 min before the probe test.

### Viral detection

For mCherry fluorescent protein detection procedure, mice were deeply anesthetized and transcardially perfused with 40 ml of saline 0.9% solution followed by 40 ml of paraformaldehyde solution 4% in PBS (PFA). Brains remained in PFA 4% for 24 h before being transferred to sucrose solution 30% for additional 24 h. Coronal sections of 40 μm were cut using a freezing microtome (Leica Microsystems), mounted on slides at 80 mm intervals (one out of every three sections) and coverslipped with Fluoromount with DAPI (Sigma-Aldrich). mCherry fluorescent protein expression was detected under a microscope (Leica DMI6000). Microphotographs of each section where mCherry fluorescent signal was seen were taken at 2.5× magnification.

**Figure 1.**
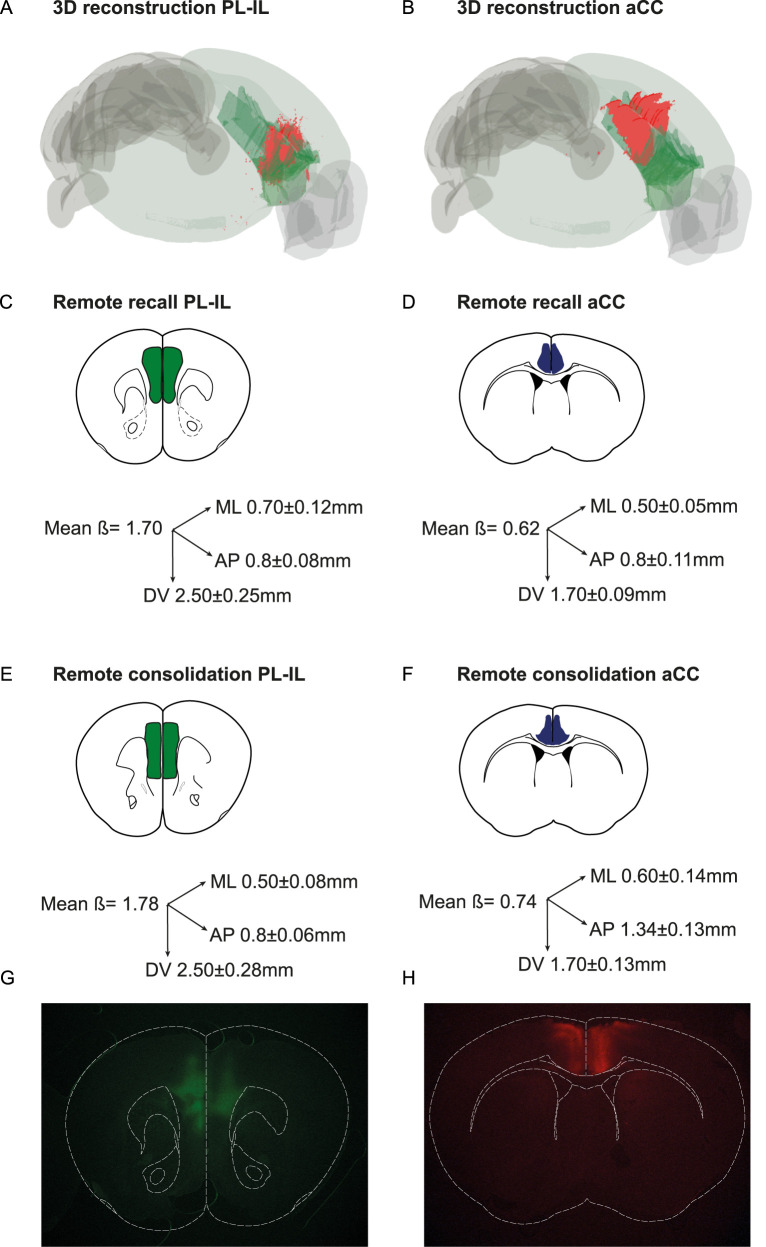
Extension of the fluorescent signal in the PL–IL and in the aCC in the different experiments. 3D reconstruction of the fluorescent in a representative mouse from the PL–IL (***A***) and from the aCC experiment (***B***). Schematic representing the fluorescent signal and the extension of the labeling on the three axes for the different experiments. ***C***, PL–IL and (***D***) aCC labeling in the recall study; (***E***) PL–IL and (***F***) aCC labeling in the consolidation experiments. *β* indicates the AP coordinate (distance from the bregma according to Franklin and Paxinos, 1997) of the maximal extension of the fluorescent labeling; number on the axes indicates the average extension of the labeling on the ML, AP, and DV axes (mean ± SEM) for the animals included in the different groups. Microphotographs showing fluorescent labeling in the PL–IL (***G***) and in the aCC cortices (***H***). See Extended Data Figures 1-1 and 1.2 for more details.

Mice were included in the experimental group of interest only when mCherry fluorescent signal was inside the target location for at least 70% of the total surface. The analysis was made in the section with the most diffuse signal, by means of area (mm^2^) of fluorescence diffusion. Moreover, we calculated the total surface of viral diffusion, to exclude animals that presented >30% of the total fluorescent signal inside the adjacent cortical regions. To this aim, we assigned a “distance from bregma” (*β*) value to each sections according to the mouse brain atlas (Franklin and Paxinos, 1997). Thereafter we averaged the *β* values corresponding to the section of maximal expression for each experimental condition (consolidation and retention). In the same section, we also measured the mediolateral (ML) and dorsoventral (DV) extension of the signal. These measurements were averaged across all mice within each experimental condition to determine the mean viral expression extension on the ML and DV axes. Finally, we calculated the extension of viral infection along the anteroposterior (AP) axis by identifying the first and the last section displaying fluorescence signal (Fig. 1*C*–*F*).

10.1523/ENEURO.0192-24.2024.f1.1 Figure 1-1. **AAV-Syn::mCherry-2A-HA-hM4Di virus diffusion for the remote retention experiments.** Schematic representation of mCherry protein expression in vmPFC **(A),** and in the aCC **(B)**. mCherry maximum extension is represented in green for each single mouse for the vmPFC group and in blue for each single mouse of aCC group. Coordinates are expressed as mm anterior to bregma. Download Figure 1-1, TIF file.

**Figure 2.**
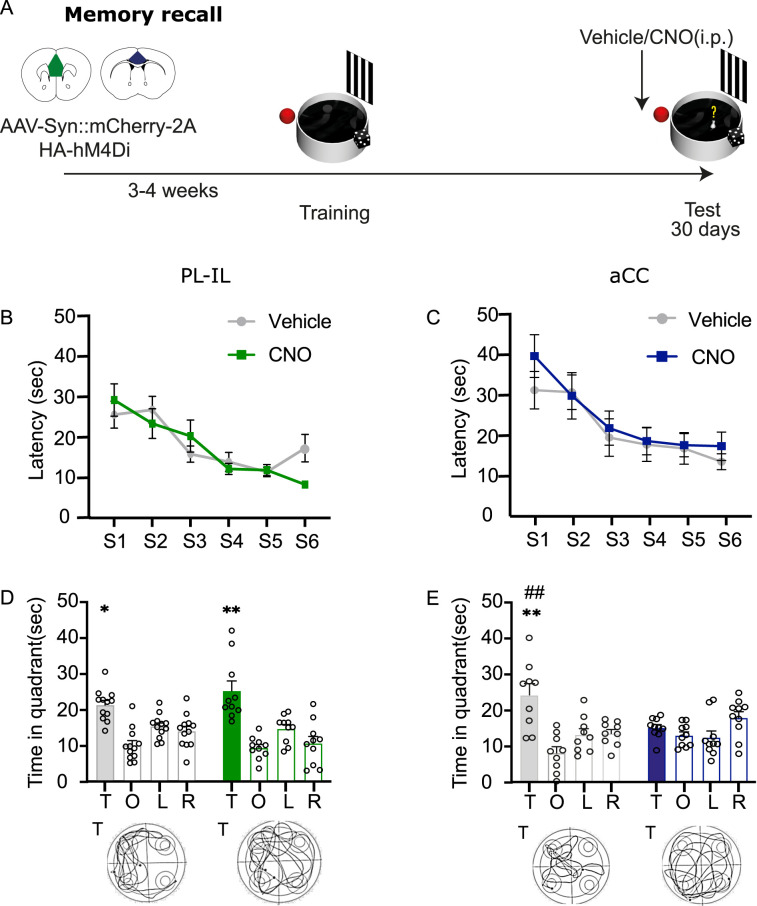
aCC but not PL–IL is required for the recall of remote spatial memories 30 d after training. ***A***, Schematic of the experimental design. ***B***, Latency (±SEM) to reach the platform during training before vehicle (*N* = 12) or CNO (*N* = 10) administrations in the PL–IL-infected groups. ***C***, Latency (±SEM) to reach the platform during training before vehicle (*N* = 9) or CNO (*N* = 10) administrations in the aCC-infected groups. ***D***, The ability to recall the correct quadrant was not affected by pretest systemic CNO (3 mg/Kg, i.p.) administrations in mice infected in the PL–IL as compared with saline. ***E***, Pretest CNO administrations impaired ability to recall the platform location as compared with saline in mice infected in the aCC. Histograms represent the mean of distance traveled in each quadrant: T, target; O, opposite; R, right; L, left. Bottom panels, Representative track plots from vehicle and CNO animals in the two experiments. *p ≤ 0.05; **p ≤ 0.01 versus target quadrant (within group); ^##^*p *≤ 0.01 target versus target quadrant (between groups; Tukey, HSD). See Extended Data Figure 2-1 for more details.

10.1523/ENEURO.0192-24.2024.f1.2 Figure 1-2 **AAV-Syn::mCherry-2A-HA-hM4Di virus diffusion for the recent and remote consolidation experiments.** Schematic representation of mCherry protein expression in vmPFC **(A),** and in the aCC **(B)**. mCherry maximum extension is represented in green for each single mouse for the vmPFC group and in blue for each single mouse of aCC group. Coordinates are expressed as mm mm anterior to bregma bregma. Download Figure 1-2, TIF file.

These analyses were performed using ImageJ (NIH). To create the 3D representation of the viral diffusion, we used one representative animal per group (consolidation experiment and retention experiment) and applied the QUINT protocol workflow for 3D images (Yates et al., 2019; Fig. 1*A*,*B*).

### Data collection and statistical analysis

Several parameters of behavioral performance were recorded. During training sessions, path length and latency to mount onto the platform were recorded in each trial. During the probe test, we measured the time spent and the distance traveled in the four quadrants and the number of annulus crossings, i.e., the number of times a mouse crossed an ideal circle (14 cm of diameter) located around each possible platform positions in the four quadrants. The average swimming speed was also calculated. All data are represented as mean ± standard error (SEM). MWM experiments were analyzed using a two-way repeated–measure ANOVA with treatment (two levels, vehicle and CNO) as between group factor and sessions (six levels, Sessions 1–6) or quadrants (four levels, target, opposite, right and left quadrants) as repeated measures, respectively, for the training and the probe test. When the interaction between factors was not significant, a one-way repeated–measure ANOVA with the six sessions or the four quadrants as repeated measures was used on each group independently. Tukey’s honest significant difference (HSD) test was used for post hoc comparison.

## Results

### Assessment of viral infection in the PL–IL and in the aCC

The neuroanatomical classification of the PL–IL and the aCC was delineated accordingly to the Franklin and Paxinos mouse brain atlas (Franklin and Paxinos, 1997) and the Allen Brain Atlas (Wang et al., 2020). The PL–IL included the IL, the PL, and anterior component of the cingulate cortex (Cg1); the aCC included the posterior component of the Cg1 and the Cg2. As represented in the 3D reconstruction of the fluorescent signal in two representative mice from the PL–IL and aCC groups, the infected area was restricted to two distinct and nonoverlapping regions (Fig. 1*A*,*B*). As shown in Figure 1, *C* and *E*, extension of the fluorescent labeling in the mice included in the two PL–IL experiments was highly superimposed (Fig. 1*C*,*E*,*G*); similar fluorescent labeling was also observed when comparing spreading in the two aCC groups (Fig. 1*D*,*F*,*H*). Finally, it should be emphasized that quantification of AP viral diffusion in the PL–IL and in the aCC demonstrates no overlap between the infected areas. Extended Data Figures 1-1 and 1-2 show the schematic representation of the area with the most diffuse fluorescent signal for all the mice included in the experiments.

### Functional dissociation of aCC and PL–IL in remote memory recall

To determine the role of these two different cortical modules in the recall of remote spatial memories, we performed a pretest chemogenetic inhibition of the PL–IL and the aCC 30 d after training in the MWM in two different groups of mice (Fig. 2*A*). As represented in Figure 2, *B* and *C*, during training the latency to reach the platform similarly decreased across the six sessions in the two experiments for both experimental groups (PL–IL: two-way repeated–measure ANOVA, session, *F*_(5,100)_ = 13.10; *p* < 0.0001; treatment, *F*_(1,20)_ = 0.2140; *p* = 0.648; session × treatment, *F*_(5,100)_ = 1.772; *p* = 0.125; aCC: two-way repeated–measure ANOVA, session, *F*_(5,85)_ = 12.65, *p* < 0.0001; treatment, *F*_(1,17)_ = 0.3698; *p* = 0.5512; session × treatment, *F*_(5,85)_ = 0.5212; *p* = 0.7596). The one-way repeated–measure ANOVA revealed a significant effect of the session for both vehicle and CNO groups in PL–IL and aCC (PL–IL vehicle, *F*_(5,55)_ = 6.732; *p* < 0.0001; PL–IL CNO, *F*_(5,45)_ = 7.724; *p* < 0.0001; aCC: vehicle, *F*_(8,40)_ = 3.128; *p* = 0.0018; aCC CNO, *F*_(5,45)_ = 8.931; *p* < 0.0001) demonstrating that the animals were able to learn the task and there were no differences between groups before treatment (Fig. 2*B*,*C*).

10.1523/ENEURO.0192-24.2024.f2.1 Figure 2-1 **Effects of pre-test loss-of-function manipulations of the vmPFC or the aCC on probe test at remote time points. (A) (left panel)** Mean distance travelled (m) ± SEM on probe test for vmPFC group [ANOVA of quadrant F_(1,20)_= 43.67, p < 0.0001; treatment F_(1,20)_= 0.016, p = 0.919; session x treatment F_(1,20)_= 0.6562, p = 0.427]. **(center panel)** Mean annulus entries ± SEM on probe test for vmPFC group [ANOVA of quadrant F_(1,20)_= 27.76, p < 0.0001; treatment F_(1,20)_= 0.394, p = 0.537; session x treatment F_(1,20)_= 1.11, p = 0.304]. **(right panel)** Mean of speed (sec) on probe test for vmPFC group [t_(20)_= 1.503, p = 0.14]. **(B) (left panel)** Mean distance travelled (m) ± SEM on probe test for aCC group [ANOVA of quadrant F_(1,17)_= 10.54, p < 0.0004; treatment F_(1,17)_= 0.0159, p = 0.901; session x treatment F_(1,20)_= 2.872, p = 0.108]. **(center panel)** Mean annulus entries ± SEM on probe test for aCC group [ANOVA of quadrant F_(1,17)_= 9.61, p < 0.0006; treatment F_(1,17)_= 0.381, p = 0.544; session x treatment F_(1,20)_= 4.126, p = 0.058]. **(right panel)** Mean of speed (sec) on probe test for aCC group [t_(17)_= 1.343, p = 0.19]. Target quadrant (T); Other Quadrants (OQ). ** p≤ 0.01; * p≤ 0.05 vs target quadrant (within group). Download Figure 2-1, TIF file.

Interestingly administrations of CNO before the probe test performed 30 d after training revealed a dissociation in the role of the PL–IL and the aCC in the ability to retrieve information relative to the platform location (Fig. 2*D*,*E*). As shown in Figure 2*D*, mice expressing hM4Di in the PL–IL when administered before the probe test with CNO intraperitoneally were able to correctly locate the platform similarly to saline-injected controls. The two-way ANOVA analysis did not highlight any differences in the time spent in the target quadrant for PL–IL groups (quadrant, *F*_(3,60)_ = 23.20; *p* < 0.0001; treatment, *F*_(1,20)_ = 0.3237; *p* = 0.5757; quadrant × treatment, *F*_(3,60)_ = 1.556; *p* = 0.2094). Further analysis confirmed that both vehicle and CNO groups were able to correctly remember the platform location (one-way repeated–measure ANOVA, vehicle, *F*_(3,33)_ = 11.21; *p* < 0.0001; CNO, *F*_(3,27)_ = 11.86; *p* < 0.0001; Fig. 2*D*). These findings were confirmed by the analysis of the distance traveled (Extended Data Fig. 2-1*A*) as well as by the analysis of the annulus frequency that is often considered directly linked to the acquisition of the correct spatial location of the platform (Lassalle et al., 2000; Ferretti et al., 2005; Extended Data Fig. 2-1*A*).

On the contrary, pretest CNO-injected mice in the aCC group showed an impaired ability to recall the platform location compared with control mice (two-way ANOVA repeated measures, quadrant preference, *F*_(3,51)_ = 8.0; *p* = 0.0002; treatment *F*_(1,17)_ = 1.6; *p* = 0.216; quadrant × treatment, *F*_(3,51)_ = 5.251; *p* = 0.0034; Fig. 2*E*). The analysis of distance traveled and annulus frequency in the different quadrants confirmed impairment of the CNO-injected mice compared with that of vehicle-injected controls (Extended Data Fig. 2-1*B*). The analysis of the mean speed during the probe test did not reveal any difference between the vehicle and CNO groups in the two experiments (Extended Data Fig. 2-1*B*).

Overall these results are in line with previous findings (Maviel et al., 2004; Teixeira et al., 2006) demonstrating the role of the PFC in the recall of remote spatial memory, but they also provide new insight into the functional role of the two cortical modules, demonstrating a functional dissociation between PL–IL and aCC and establishing that only the aCC is involved in the retrieval of remote spatial memories.

### Both the PL–IL and the aCC are required for the consolidation of remote but not recent spatial memories

The establishment of stable remote memory traces requires a consolidation process that starts immediately after encoding (McGaugh, 2000). However, experimental evidence investigating the role of the PFC in the consolidation of the memory trace, based mainly on fear memory tests, is contradictory and leaves doubts on when this brain region is engaged and whether its role is limited to the consolidation of remote memories or it is also functional to the maintenance of memories at shorter intervals (Izquierdo et al., 2007; Restivo et al., 2009; Leon et al., 2010; Lesburguères et al., 2011; Bero et al., 2014; Torres-García et al., 2017; Vetere et al., 2017; Pereira et al., 2019). Therefore, next we asked whether immediate post-training loss-of-function manipulations of the PFC could impair consolidation of spatial memory at early and late time points by using chemogenetics. Moreover, in light of the functional dissociation between the PL–IL and the aCC found in the recall of spatial information, we contrasted the two brain regions to see whether a similar dissociation could be observed also on the consolidation.

**Figure 3.**
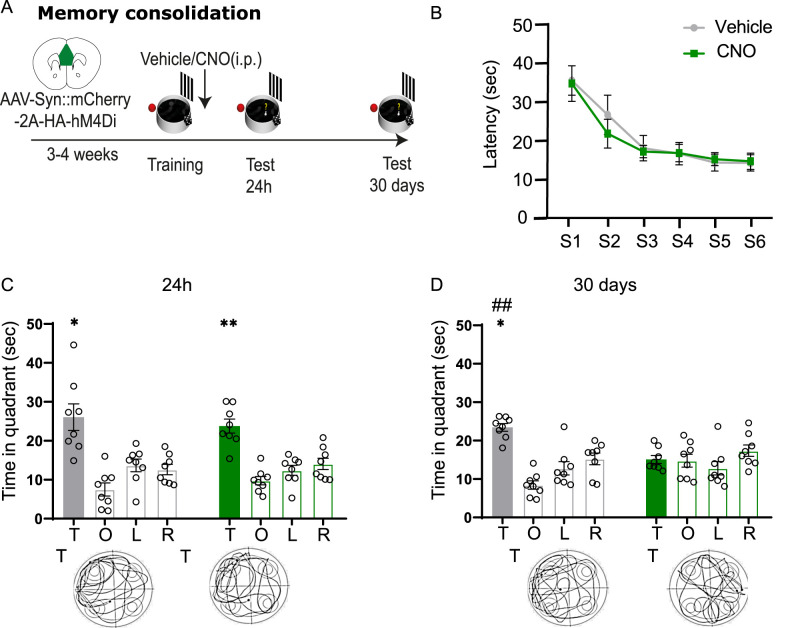
PL–IL activation is required to consolidate remote but not recent spatial memories. ***A***, Schematic of the experimental design. ***B***, Latency (±SEM) to reach the platform during training before vehicle (*N* = 8) or CNO (*N* = 8) post-training administrations in the PL–IL-infected groups. ***C***, Post-training systemic administrations of CNO did not affect mice ability to recall the correct platform location 24 h after training compared with vehicle controls. ***D***, Mice administered post-training with CNO showed impaired performance on the probe test 30 d after training. Histograms represent the mean of distance traveled in each quadrant: T, target; O, opposite; R, right; L, left. Bottom panels, Representative track plots from vehicle and CNO animals in the two experiments. *p ≤ 0.05; **p ≤ 0.01 versus target quadrant (within group); ^##^*p *≤ 0.01 target versus target quadrant (between groups; Tukey, HSD). See Extended Data Figure 3-1 for more details.

In the first experiment, a group of mice, virally injected in PL–IL, was trained in the MWM and immediately post-training administered intraperitoneally with either vehicle or CNO (Fig. 3*A*). No differences were found in the training of the two groups (two-way repeated–measure ANOVA, sessions, *F*_(5,70)_ = 14.76; *p* < 0.0001; treatment, *F*_(1,14)_ = 0.1355; *p* = 0.7183; session × treatment, *F*_(5,70)_ = 0.2505; *p* = 0.9382) that progressively reduced the time to find the platform during successive sessions (one-way repeated–measure ANOVA, vehicle, *F*_(5,35)_ = 6.601; *p* = 0.0002; CNO, *F*_(5,35)_ = 9.041; *p* < 0.0001) confirming the ability of the mice to properly acquire the task before treatment (Fig. 3*B*).

10.1523/ENEURO.0192-24.2024.f3.1 Figure 3-1 **Effects of immediate post-training loss-of-function manipulations of the vmPFC on probe test at recent (24  h) and remote (30d) time points. (A)** Probe trial 24 hour after training of mice infected in the vmPFC and administered immediately post-training with vehicle or CNO. **(left panel**) Mean of distance travelled (m) ± SEM [ANOVA of quadrant F_(1,14)_= 26.82, p < 0.0001; treatment F_(1,14)_= 0.158, p = 0.696; session x treatment F_(1,14)_= 0.46, p = 0.506]; (**center panel**) Mean annulus entries ± SEM [ANOVA of quadrant F_(1,14)_= 22.84, p < 0.001; treatment F_(1,14)_= 0.277, p = 0.606; session x treatment F_(1,14)_= 0.300, p = 0.592]; (**right panel**) Mean of speed (sec) ± SEM [t_(14)_= 1.979, p = 0.84] on probe test 24  h after training for vmPFC infected groups. **(B).** Probe test 30 days after training of vmPFC infected mice administered with vehicle or CNO immediately after training. (**left panel**) Mean of distance travelled (m) ± SEM [ANOVA of quadrant F_(1,14)_= 34.38, p = 0.00017; treatment F_(1,14)_= 7.16, p = 0.018; session x treatment F_(1,14)_= 27.42, p = 0.00013]; (**center panel**) Mean annulus entries ± SEM [ANOVA of quadrant F_(1,14)_= 36.38, p < 0.001; treatment F_(1,14)_= 7.92, p = 0.014; session x treatment F_(1,14)_= 24.02, p < 0.001]. (**right panel**) Mean of speed (sec) ± SEM [t_(14)_= 0.88, p = 0.39]. Target quadrant (T); Other Quadrants (OQ). *p≤ 0.05; ** p≤ 0.01 vs target quadrant (within group); ## p≤ 0.01 target vs target quadrant (between groups) (Tukey, HSD). Download Figure 3-1, TIF file.

When we analyzed the time spent by the mice in the different quadrants during the probe test 24 h after training, we found that both vehicle- and CNO-injected mice spent significantly more time in the target quadrant (two-way repeated–measure ANOVA of quadrant, *F*_(3,42)_ = 21.61; *p* < 0.0001; treatment, *F*_(1,14)_ = 1.224; *p* = 0.2873; quadrant × treatment, *F*_(3,42)_ = 0.5290; *p* = 0.6648) regardless of the treatment (one-way repeated–measure ANOVA, vehicle, *F*_(3,21)_ = 9.878; *p* = 0.0003; CNO, *F*_(3,21)_ = 13.85; *p* < 0.0001; Fig. 3*C*). On the other hand, CNO-injected mice showed a clear impairment in remembering the platform location 30 d after training compared with controls (two-way repeated–measure ANOVA, quadrant, *F*_(3,42)_ = 8.788; *p* = 0.0001; treatment, *F*_(1,14)_ = 1.690; *p* = 0.2146; quadrant × treatment, *F*_(3,42)_ = 6.917; *p* = 0.0007; Fig. 3*D*). The results were confirmed by the additional analyses on the distance traveled and the annulus entries either 24 h or 30 d after training (Extended Data Fig. 3-1). Once again, the results on the mean speed during the probe test confirmed that there were no differences between vehicle- and CNO-treated groups (Extended Data Fig. 3-1).

**Figure 4.**
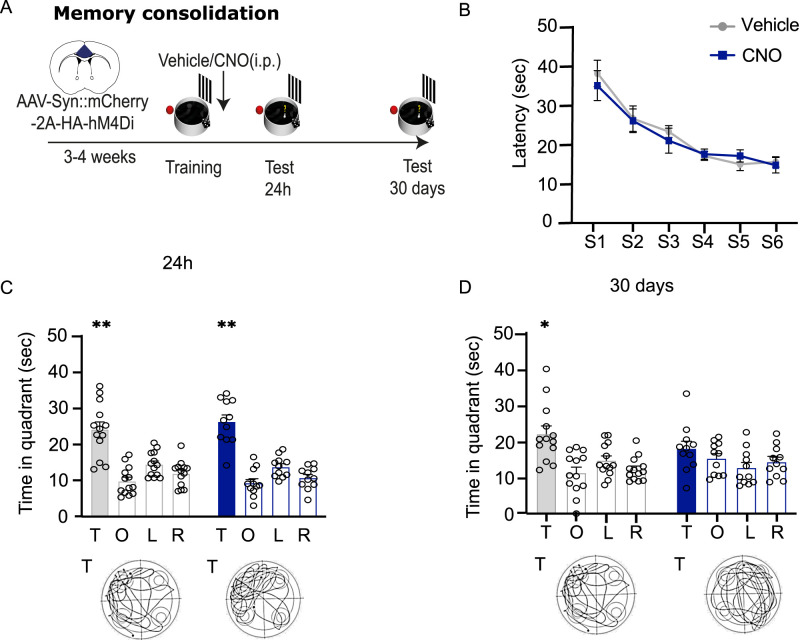
aCC activation is required to consolidate remote but not recent spatial memories. ***A***, Schematic of the experimental design. ***B***, Latency (±SEM) to reach the platform during training before vehicle (*N* = 8) or CNO (*N* = 8) post-training administrations in the aCC-infected groups. ***C***, Post-training systemic administrations of CNO did not affect mice ability to recall the correct platform location 24 h after training. ***D***, Post-training administration of CNO impaired mice ability to recall the platform location 30 d after training. Histograms represent the mean of distance traveled in each quadrant: T, target; O, opposite; R, right; L, left. Bottom panels, Representative track plots from vehicle and CNO animals in the two experiments. *p ≤ 0.05; **p ≤ 0.01 versus target quadrant (within group; Tukey, HSD). See Extended Data Figure 4-1 more details.

To determine the role of the aCC in the consolidation of recent and remote spatial memory, in a second experiment we trained mice that had been virally injected in the aCC and performed immediate post-training administrations of either vehicle or CNO. We then tested them 24 h and 30 d after training (Fig. 4*A*). As for the PL–IL experiment, we did not find any difference in the learning curve of the two groups before saline or CNO administrations (two-way repeated–measure ANOVA of session, *F*_(5,110)_ = 26.39; *p* < 0.0001; treatment, *F*_(1,22)_ = 0.1514; *p* = 0.7009; session × treatment, *F*_(1,22)_ = 0.1514; *p* = 0.7009). Further analysis confirmed that both groups were able to acquire information relative to the platform location, progressively reducing the time to reach the platform over successive sessions (one-way repeated–measure ANOVA, vehicle, *F*_(5,60)_ = 18.64; *p* < 0.0001; CNO *F*_(5,50)_ = 9.320; *p* < 0.0001; Fig. 4*B*). When tested for recent memory 24 h after training, the mice in both vehicle and CNO groups were able to remember the platform location (two-way repeated–measure ANOVA, quadrants, *F*_(3,66)_ = 41.70; *p* < 0.0001; treatment, *F*_(1,22)_ = 2.592; *p* = 0.1217; quadrant × treatment, *F*_(3,66)_ = 0.4726; *p* = 0.7024), spending significantly more time in the target quadrant (one-way repeated–measure ANOVA, vehicle, *F*_(3,36)_ = 16.43; *p* < 0.0001; CNO, *F*_(3,30)_ = 27.38; *p* < 0.0001; Fig. 4*C*). On the contrary, when testing was performed 30 d after training, only mice administered post-training with vehicle were able to correctly recall the platform location (two-way repeated–measure ANOVA of quadrant, *F*_(3,66)_ = 6.549; *p* = 0.0006; treatment, *F*_(1,22)_ = 0.5776; *p* = 0.4553; quadrant × treatment, *F*_(3,66)_ = 2.010; *p* = 0.1210; one-way repeated–measure ANOVA of controls, *F*_(3,36)_ = 7.099; *p* = 0.0007; CNO, *F*_(3,30)_ = 1.531; *p* = 0.2267; Fig. 4*D*). Analysis on the distance traveled and the annulus frequency confirmed that only control animals were able to correctly remember the platform location (Extended Data Fig. 4-1). Analysis of mean speed did not reveal any difference between the two groups (Extended Data Fig. 4-1).

10.1523/ENEURO.0192-24.2024.f4.1 Figure 4-1 **Effects of immediate post-training loss-of-function manipulations of the aCC on probe test at recent (24  h) and remote (30d) time points. (A**) Effect of immediate post-training administrations of vehicle or CNO on probe trial 24 hours after training in aCC infected mice**. (left panel**) Mean of distance travelled (m) ± SEM [ANOVA of quadrant F_(1,22)_= 63.75, p < 0.001; treatment F_(1,22)_= 3.21, p = 0.087; session x treatment F_(1,22)_= 2.405, p = 0.135]; (**center panel**) Mean annulus entries ± SEM [ANOVA of quadrant F_(1,22)_= 52.07, p < 0.001; treatment F_(1,22)_= 0.09, p = 0.765; session x treatment F_(1,22)_= 0.127, p = 0.724]; (**right panel**) Mean of speed (sec) ± SEM [t_(22)_= 1.041, p = 0.3] (**B)** Probe trial 30 days after training of aCC infected mice administered immediately post-training administrations with vehicle or CNO. **(left panel**) Mean of distance travelled (m) ± SEM [ANOVA of quadrant F_(1,22)_= 13.76, p = 0.0012; treatment F_(1,22)_= 1.55, p = 0.226; session x treatment F_(1,22)_= 0.78, p = 0.38]; (**center panel**) Mean annulus entries ± SEM [ANOVA of quadrant F_(1,22)_= 5.16, p = 0.033; treatment F_(1,22)_= 0.0002, p = 0.99; session x treatment F_(1,22)_= 0.71, p = 0.408]; (**right panel**) Mean of speed (sec) ± SEM [t_(22)_= 1.993, p = 0.058]. Target quadrant (T); Other Quadrants (OQ). *p≤ 0.05; ** p≤ 0.01 vs target quadrant (within group); ## p≤ 0.01 target vs target quadrant (between groups) (Tukey, HSD). Download Fig.4.1, TIF file.

These findings demonstrate that the PFC is involved in the early phases of the consolidation of remote but not recent spatial memories; moreover they revealed that the two cortical modules play a similar role in this process.

## Discussion

Contrasting the two components of the PFC, the aim of this study was twofold: to investigate their relative contribution to the recall of remote spatial memory and to the consolidation of both recent and remote memories. The results demonstrated an interesting dissociation between the two components. Loss-of-function manipulation of the aCC, but not the PL–IL, impaired recall of remote spatial memory; however, both subdivisions contributed to its remote consolidation.

Current hypotheses on memory consolidation suggest that, hours after learning, molecular changes in the hippocampus lead to synaptic strengthening within this region (Kandel et al., 2014; Dudai et al., 2015). Over weeks, the role of the hippocampus degrades, and cortical structures become more important (Frankland and Bontempi, 2005). Our results confirm this view, demonstrating that chemogenetic inhibition of the aCC before testing impairs the ability to recall the correct platform location 30 d after the last training session. Interestingly, this effect was specific to the aCC; in fact, when the same manipulation was performed in the PL–IL, the animals were perfectly able to locate the platform. It should be mentioned that, in both cases, we performed systemic administrations of CNO, but the lack of effect in the PL–IL group excludes the possibility that the effects observed in the aCC group depend on CNO alone.

IEG expression has been found to increase after remote memory recall in both the PL–IL and the aCC for different kinds of information (Frankland et al., 2004; Maviel et al., 2004; Lopez et al., 2012). However, causal interrogation of their role in the recall of remote memory in different tasks suggests a functional distinction between the two components. For example, inactivation of the aCC impairs remote recall in both fear conditioning (Frankland et al., 2004) and the radial arm maze (Maviel et al., 2004). On the contrary, the PL–IL seems to be involved only in the recall in the radial arm maze but not in contextual fear conditioning (Frankland et al., 2004; Maviel et al., 2004). Although the findings reported in the present study are in line with previous reports demonstrating the impairing effect of loss-of-function manipulation of the aCC in the recall of remote memories in the MWM (Teixeira et al., 2006; Wartman et al., 2014), to our knowledge, this is the first direct comparison of the effects of loss-of-function manipulation of the two PFC components in the MWM, demonstrating a dissociation between the two PFC subdivisions similar to what has been found for contextual fear conditioning (Frankland et al., 2004). Based on the present findings alone, it is difficult to explain these differences. Nevertheless, it is worth noting that differences in c-Fos expression in the aCC and the PL–IL have been observed at remote time points in the MWM when comparing task conditions affecting memory strength (Lopez et al., 2012). Moreover, loss-of-function manipulations of the PL–IL have been shown to improve performance in the MWM at remote time points only when there is the need to solve conflicting information between new and stored information on the platform location (Richards et al., 2014). These observations suggest that the PL–IL might come into play only when remotely acquired information, progressively fading away, needs to compete for performance with strong newly acquired memories (Eichenbaum, 2017). Although very speculative, this view could explain the discrepancy reported in the literature as well as our finding where there is no competition between new and remote memories in the probe trial.

In the second experiment, we contrasted the two components of the PFC in the consolidation of spatial memories at recent and remote time points. Surprisingly in contrast to the dissociation observed in the recall of remote memories, we found that the two PFC compartments behave in a similar way. In fact, immediate post-training manipulations in either the PL–IL or the aCC impaired recall at late time points leaving unaffected the ability to correctly locate the platform 24 h after training. A caveat to be considered in our experimental design is that all groups of mice were tested at both time points, 24 h and 30 d after training. It seems unlikely that the impairing effect observed might be due to the procedure used. Single exposure to the maze without the platform did not impair the control mice's ability to locate the platform on the second probe trial, 30 d after training. Interactions between the first probe trial and the treatment seem also unlikely, as CNO was administered immediately after training and the first probe trial was conducted 24 h later, when the animals were no longer under the influence of the drug. Therefore, the simplest explanation of the results presented is an impairing effect of the manipulations of the two components of the PFC on the consolidation of remote, but not recent, spatial memory. These findings raise several interesting issues. First, they provide new evidence that neuronal activity in the PFC immediately after training is required for the consolidation of remote but not recent spatial memory as assessed in the MWM. Studies investigating the role of the PFC in the consolidation of memory are primarily based on aversively motivated tasks (Izquierdo et al., 2007; Bero et al., 2014; Torres-García et al., 2017; Vetere et al., 2017; Pereira et al., 2019). This is mainly due to the difficulty of separating the encoding and the consolidation phase in spatial learning tasks requiring training over multiple days, where consolidation occurs repeatedly at the end of each training period. In the present study, we used a massed version of the MWM that allows manipulation after a single training, lasting ∼1 h, at the early stages of the consolidation phase. It should be mentioned that a similar experimental strategy has been previously adopted by Leon et al. (2010). They reported, on the one hand, a learning-induced increase in ERK1/2 phosphorylation in the hippocampus but not in the PFC immediately after training. On the other hand, they observed an impairing effect of post-training PFC administrations of the mitogen-activated protein kinase (MAPK)/ERK inhibitor (U0126) on the ability to correctly locate the platform 24 h after training (Leon et al., 2010). Although there is an apparent discrepancy between the two observations, the impairing effects induced by post-training administrations of U0126 in the PFC seem in contrast with the lack of effect we report in the present study. It is worth noting that contrasting results on the role of PFC in the consolidation of recent memory have been reported also in other learning tasks such as one-trial inhibitory avoidance (Izquierdo et al., 2007; Torres-García et al., 2017), fear conditioning (Bero et al., 2014; Vetere et al., 2017; Pereira et al., 2019) and odor discrimination tasks (Tronel and Sara, 2003; Lesburguères et al., 2011), depending on the PFC module manipulated or the time window chosen for the manipulation. Further studies will be needed to better understand the contribution of PFC and its subdivisions in the consolidation of recent memories.

Another intriguing issue is the similar effect induced by loss-of-function manipulation of the two PFC components in the consolidation of remote spatial memory, despite their functional differences in remote recall. Previous studies have generally excluded early engagement of different PFC components immediately after training, based on the lack of change in neuronal activity observed through IEG expression after probe tests at late time points (Frankland et al., 2004; Maviel et al., 2004). Our findings support the notion that activity in both subdivisions of the PFC is required early after learning to promote the formation of stable memory traces that can be recalled at late time points. Although direct comparison between whole brain region loss-of-function manipulation, such as the one we used in the present study, and the engram cells approach is difficult, intriguingly, utilizing activity-dependent labeling to track the activation pattern of engram cells in the PFC after training and their causal role in fear conditioning has led to similar conclusions (Kitamura et al., 2017; DeNardo et al., 2019). What was particularly surprising to us was the dissociation observed in the PL–IL, which we found to impair the consolidation of remote spatial memories but not their recall. Therefore, interestingly, the PL–IL appears to be involved solely in the consolidation of remote spatial memory traces, differently from both the HPC (Riedel et al., 1999) and the aCC, which are engaged in both the consolidation and recall at recent or remote time points.

The evidence presented in this study establishes that neuronal activity in both the PFC subregions is necessary immediately after training in the MWM to consolidate spatial information needed for correctly locating the platform at remote, but not recent, time points. Moreover, we demonstrated the role of the aCC, but not the PL–IL, in the recall of the platform location at remote time points. This suggests that, after training, the activity of the PL–IL gradually declines, and the aCC takes over in retaining the information playing an active role in their remote recall. It could be suggested that spatial memory immediately after encoding engages a wide network of brain regions that include the HPC (Riedel et al., 1999), the PL–IL, the aCC, and possibly other brain regions to initiate the consolidation process. Once the memory trace is well consolidated, the information is stored over time in the aCC; then the PL–IL comes into play and participates to the recall only if there is a competition for the performance between old and new memories (Richards et al., 2014; Eichenbaum, 2017). It has been suggested that the HPC to PFC projections are important in controlling the formation of stable memory traces (Barker et al., 2017). Although the connectivity of the two PFC subdivisions is not completely segregated, the PL–IL is better characterized by limbic afferent projections and dopaminergic modulation (Heidbreder and Groenewegen, 2003; Uylings et al., 2003; Le Merre et al., 2021). It would therefore be intriguing to explore whether PL–IL might play a role in the selection of relevant information to be stored in the aCC gating HPC input to the PFC. Such investigations could shed further light on the complex dynamics underlying memory consolidation and recall processes.

## Synthesis

Reviewing Editor: Laura Bradfield, University of Technology Sydney Decisions are customarily a result of the Reviewing Editor and the peer reviewers coming together and discussing their recommendations until a consensus is reached. When revisions are invited, a fact-based synthesis statement explaining their decision and outlining what is needed to prepare a revision will be listed below. The following reviewer(s) agreed to reveal their identity: Samantha White. Note: If this manuscript was transferred from JNeurosci and a decision was made to accept the manuscript without peer review, a brief statement to this effect will instead be what is listed below.

Thank you for your revisions and rebuttal for this manuscript, and my apologies for the delay in re-review. The manuscript is much improved but there are still some minor outstanding issues. In particular, as noted in detail by reviewer #2, it would be ideal to see the data from the preliminary study, even briefly. If the authors do not wish to share these data then, at minimum, I would request that the authors add information in the main text regarding the fact that such a preliminary study was done (with data not shown) and did not provide any evidence suggesting an influence on results from multiple rounds of testing. As suggested by Reviewer #2, could the authors please include the details of this study when detailing this (possibly in the methods section in which the procedure is described - as rationale for the current procedure). In addition, please can the authors add a sentence or two to the discussion regarding the limitations of the study and the possible confound, as described by Reviewer #2.

Reviewer #1

Overall, the two studies in this manuscript complement each other and reveal interesting contributions of these two cortical regions in spatial memory recall and consolidation at different timepoints. The rationale for the study is well defined, the anatomy is clearly described (and shown in Figure 1), and the study is presented simply which makes it easy to follow. The revisions made to the manuscript addressed the major concerns during peer review.

Reviewer #2

My points to the rebuttal:

1. Author: We agree with the referee. As mentioned in our response to the editor, this was also our concern when we started the study. Our choice of testing the animals twice instead of using two different experimental groups was based on the results of a preliminary experiment (data not shown) that did not highlight any major difference between mice tested once and mice tested twice. As mentioned in our response to the editor, the behavior of mice included in the experimental groups confirmed this preliminary observation.

Mine: How was your preliminary experiment done in detail? Is it done in CNO injected animals, vehicle injected animals or naïve animals? If it was done in CNO injected animals, please show the figures and statistics in supplementary. Because this information is important for the readers. If it was done in vehicle injected or naïve animals, this could not fully address the question. Because we don't know whether Treatment 1 (saline and vehicle) and Treatment 2 (the probe tests) has interaction. There is a possibility that CNO could amplify the extinction effect, although the extinction is too subtle to be detected in the vehicle injected or naïve animals. Thus, the best way is to conduct the 24h and 30-day tests in separate group of mice. If it is not possible to redo the experiments, the author should add some sentences in the discussion part to remind the readers the defects of the experiment design and the possibility that probe test could be a potential extinction process and amplified by deactivation of aCC and mPFC.

2. Although I was able to find the edits the author mentioned, but I was confused by the line number indicated. For example, in rebuttal 2.2), "line 64" does not match "pg. 4, line 2". This happens nearly in all points in the rebuttal. And in rebuttal 2.11), line 283-235 is apparently a writing mistake.
